# SEX-SPECIFIC BIOMARKER PREDICTION OF FEMORAL NECK BONE LOSS AFTER HIP FRACTURE

**DOI:** 10.1093/geroni/igae098.2300

**Published:** 2024-12-31

**Authors:** Alan Rathbun, Marc Hochberg, Michelle Shardell, Alice Ryan, Denise Orwig, Jay Magaziner

**Affiliations:** University of Maryland School of Medicine, Baltimore, Maryland, United States; University of Maryland School of Medicine, Baltimore, Maryland, United States; University of Maryland School of Medicine, Baltimore, Maryland, United States; University of Maryland School of Medicine, Baltimore, Maryland, United States; University of Maryland School of Medicine, Baltimore, Maryland, United States; University of Maryland School of Medicine, Baltimore, Maryland, United States

## Abstract

Hip fracture exacerbates age-related inflammation and metabolic dysfunction, which could accelerate bone loss post-fracture. This study aimed to develop biomarker prediction models for declines in femoral neck bone mineral density (BMD) after hip fracture. Participants were Caucasian men (n=99) and women (n=75) from the Baltimore Hip Studies 7th cohort with a hip fracture who were not receiving glucocorticoids, sex-hormone therapy, or bone-active medications. Data were collected at baseline (within 15 days of admission) and 2-, 6-, and 12-months later. Biomarkers were categorized into tertiles: interleukin-1 receptor antagonist (IL-1RA), interleukin-6 (IL-6), soluble tumor necrosis factor-alpha (TNF-alpha) receptor type 1, estradiol (E), testosterone (T), intact parathyroid hormone (iPTH), sex hormone binding globulin (SHBG), insulin-like growth factor-1 (IGF-1), C-terminal telopeptide of type I collagen (CTX), and procollagen type I N-propeptide (PINP). Femoral neck BMD was measured using dual energy x-ray absorptiometry. Mixed-effects models adjusted for age, height, and weight estimated 1-year changes in femoral neck BMD after hip fracture. Bone loss was classified as accelerated for intra-individual changes that exceeded the mean decline. Logistic regression models were stratified by biological sex and implemented using stepwise selection. The area under the curve (AUC) estimate among men was 0.68, and biomarkers related to bone loss were E, IGF-1, SHBG, and CTX. Similarly, the AUC metric among women was 0.69, but identified biomarkers were IL-6, iPTH, IGF-1, and SHBG. Findings indicate that both inflammatory and hormonal biomarkers relate to accelerated bone loss post-fracture; however, there are differential associations in physiological mechanisms between men and women.

